# Optimising rainfall characteristics for determining landslide thresholds

**DOI:** 10.1007/s11069-025-07835-7

**Published:** 2026-02-19

**Authors:** Himasha Abeysiriwardana, Thomas Kjeldsen, Cormac Reale

**Affiliations:** Department of Architecture and Civil Engineering, Claverton Down Campus, University of Bath, Bath, BA2 7AY UK

**Keywords:** Rainfall thresholds, Landslides, Minimum inter–event time, Bayesian inference, Data scarcity, Event rainfall–duration, Intensity–duration

## Abstract

This work contributes a new framework for establishing data-driven rainfall thresholds in high-risk, data-limited contexts. Rainfall thresholds are commonly used to characterise the precipitation needed to trigger landslides in a region. However, these empirical relationships are sensitive to the exact definition of a “rainfall event”, especially how the minimum inter-event time (MIT) and triggering event (TE) are defined. Using Bayesian inference (BI) and nonlinear least-squares (NLS) techniques, this study evaluates how variations in MIT and TE definitions affect rainfall threshold estimation, considering both Event Rainfall–Duration $\left( {E{-}D} \right)$ and Intensity–Duration $\left( {I{-}D} \right)$ spaces. The dataset includes 15-min rainfall measurements from 52 gauges recorded from 2005 to 2023, as well as a regional landslide dataset compiled from British Geological Survey records covering the South Wales coalfields. Findings reveal that *BI*-derived thresholds are more stable than *NLS*-based thresholds, showing smaller parameter changes and fewer unrealistic curves, particularly in I–D space, where *NLS* often produces near-flat thresholds. Overall, both *BI* and *NLS* approaches demonstrate their strongest performance at MIT = 48 h, emphasising the role of extended antecedent rainfall in triggering spoil tip failures. This study demonstrates how the integration of robust Bayesian methods facilitates the downscaling of global thresholds to data-scarce regions and how careful event delineation practices can improve landslide prediction.

## Highlights

Landslide rainfall thresholds were derived using Bayesian and nonlinear least-squares methods.

Rainfall–Duration (E–D) thresholds performed better than Intensity–Duration (I–D) thresholds.

Minimum inter-event time (MIT) of 48 h yielded the most robust prediction results.

The Bayesian approach generally outperformed the nonlinear least-squares method.

## Introduction

**Fig. 1:**
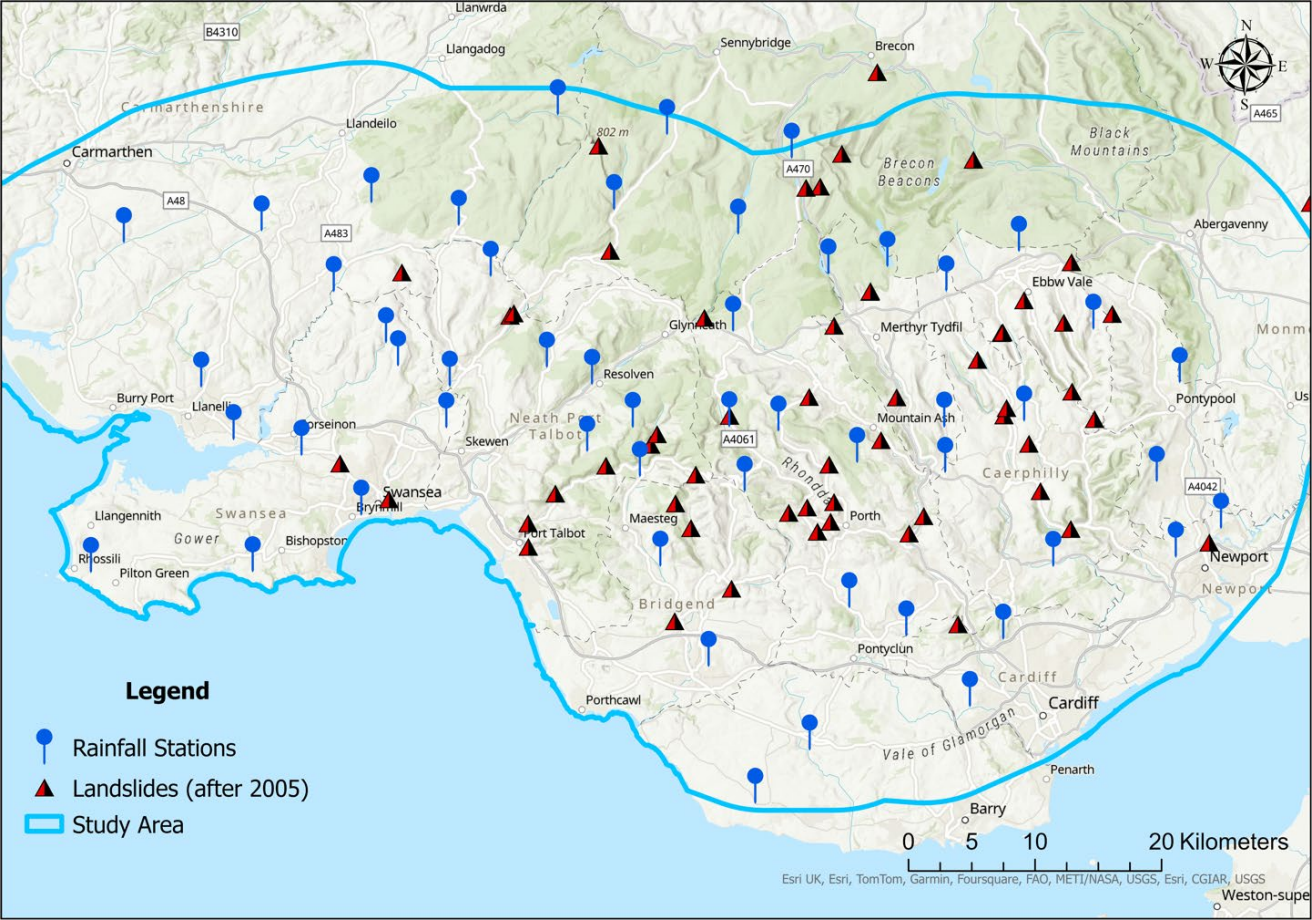
Map of study area showing locations of recorded landslides with known dates from 2005 to 2023. Source of landslide data: British Geological Survey (BGS)

Landslides are common hazards in mountainous and hilly regions worldwide resulting in numerous fatalities and considerable economic losses each year. Haque et al. (2019) estimated that landslides were the direct cause of reported 163,658 deaths across 128 countries between 1995 and 2014. Rainfall-induced slope failures are particularly common, as precipitation can destabilise soil and rock masses through infiltration, affecting pore-water pressures, and reducing soil shear strength (Zhang et al. 2011; Reale et al. 2017). Recent coal tip landslides in South Wales, most notably the 2020 Tylorstown slide triggered by Storm Dennis, have emphasised the vulnerability of spoil tip slopes in the region to heavy rainfall, reigniting memories of the traumatic 1966 Aberfan disaster (Johnes, 2000). The South Wales coalfield (Fig. 1), extend across approximately 2700 km^2^ (Robins et al. 2008)**,** and contains over 2085 disused coal tips resulting from historical end-tipping practices. Many of these tips stand at or near their angle of repose, affording minimal safety margins (Welsh Government 2022). Despite having a risk register (based on qualitative data), which identifies 350 tips across the region as being high risk (Welsh Government 2022), there remains no dedicated data-driven rainfall threshold model to support landslide prediction in this region.

Landslide prediction techniques can be broadly categorised as empirical, probabilistic and physically based methods (Berti et al. 2012). Due to their simplicity, empirical methods have seen the most application (Greco et al. 2023). In the context of rainfall induced landslides, empirical approaches establish rainfall thresholds, i.e. rainfall conditions which, if met or surpassed, are likely to induce landslides (Guzzetti et al. 2008). These models are developed by correlating past landslide events with triggering and/or predisposing precipitation conditions (Uwihirwe et al. 2020; Greco et al. 2023; Sun et al. 2024). Most regularly, empirical rainfall thresholds have been defined based on Rainfall Intensity–Duration $\left( {I{-}D} \right)$ (e.g., Caine 1980; Guzzetti et al. 2007; Iadanza et al. 2016; Marino et al. 2020; Jiang et al. 2021; Leonarduzzi et al. 2021) or Event Rainfall–Duration $\left( {E{-}D} \right)$ relationships (e.g., Peruccacci et al. 2017; Martinovic et al. 2018; Melillo et al. 2018; Abraham et al. 2021). $I{-}D$ thresholds relate mean rainfall intensity to storm duration, while $E{-}D$ thresholds account for cumulative rainfall over a defined storm duration.

A disadvantage of these empirical relationships is that they can be sensitive to the exact definition of a “rainfall event” (Melillo et al. 2015). Choices such as determining the minimum inter-event time (MIT), which is the minimum dry period between two consecutive and independent rainy periods (Iadanza et al. 2016; Wang et al. 2019; Tu et al. 2023), or determining the point in the rainfall event to relate to triggering (e.g. peak (Frattini et al. 2009) versus end (Vessia et al. 2014) of the rainfall event) can have an outsized impact on threshold generation.

While there is an established literature on rainfall thresholds (Caine 1980; Jaiswal and Westen 2009; Brunetti et al. 2010; Iadanza et al. 2016; Peruccacci et al. 2017; Piciullo et al. 2017; Martinovic et al 2018; Abraham et al. 2021), no studies have systematically tested how rainfall event characterisation (triggering and non-triggering) (Peres and Cancelliere 2018), choice of MIT, or different modelling frameworks (Brunetti et al. 2010; Rossi et al. 2017; Peres and Cancelliere 2021) influence threshold robustness. This is particularly true for regions where data are sparse or imbalanced, i.e. where landslide-triggering rainfall events are vastly outnumbered by non-triggering rainfall events.

To address these important gaps, this study systematically investigates how MIT criteria (ranging from sub-daily to multi-day), and triggering-event definitions (Peak vs. End) affect the estimation and performance of rainfall thresholds for landslide prediction in the South Wales coalfield. Both $E{-}D$ and $I{-}D$ thresholds are considered to determine how different parameter spaces capture rainfall conditions leading to slope failures. A comparison between two modelling frameworks: Bayesian Inference (BI) and Non-Linear Least-Square (NLS), is conducted to assess their effectiveness in generating reliable threshold models with pronounced class imbalance in the dataset.

The dataset compiled for this study comprises of 15-min rainfall observations from 52 automated rainfall gauges collected over the period of 2005–2023 as well as an inventory of 58 landslides in the region obtained from the British Geological Survey (BGS). By evaluating thresholds against established regional threshold models using confusion matrix-based measures (False Negative Rate, True Positive Rate, False Positive Rate, True Skill Statistic, and Area Under the Receiver Operating Characteristics Curve ), the reliability and robustness of each threshold under varying conditions are established. By comparing threshold performance across a range of MIT values, we highlight the key trade-offs in capturing short- vs. long-term antecedent rainfall. The findings offer novel insights into selecting an appropriate approach and methodological parameters (e.g., MIT, Peak vs. End, and modelling framework), thereby informing landslide early warning systems and guiding future research in threshold-based landslide forecasting.

This work contributes a framework for establishing data-driven rainfall thresholds in high-risk, data-limited contexts, demonstrating how the integration of robust Bayesian methods can allow regional downscaling of global thresholds, and how careful event-delineation practices can improve landslide prediction. Ultimately, the study’s findings offer guidance for practitioners and policymakers in South Wales and similarly constrained regions worldwide, where severe rainfall episodes can trigger catastrophic slope failures in coal tips and other marginally stable slopes.

## Study area and data collection

The study area encompasses the South Wales coalfield and adjacent areas, covering an area approximately 4600 km^2^ (see Fig. 1). The topography of the area is characterised by coastal lowlands becoming hilly inland. The elevation ranges across the study area between 0 and 900 m, with 60% of the area having elevations in excess of 100 m. Slope angle varies from 0 to 50°, with approximately 30% of the area having slope angles in excess of 8°.

**Fig. 2:**
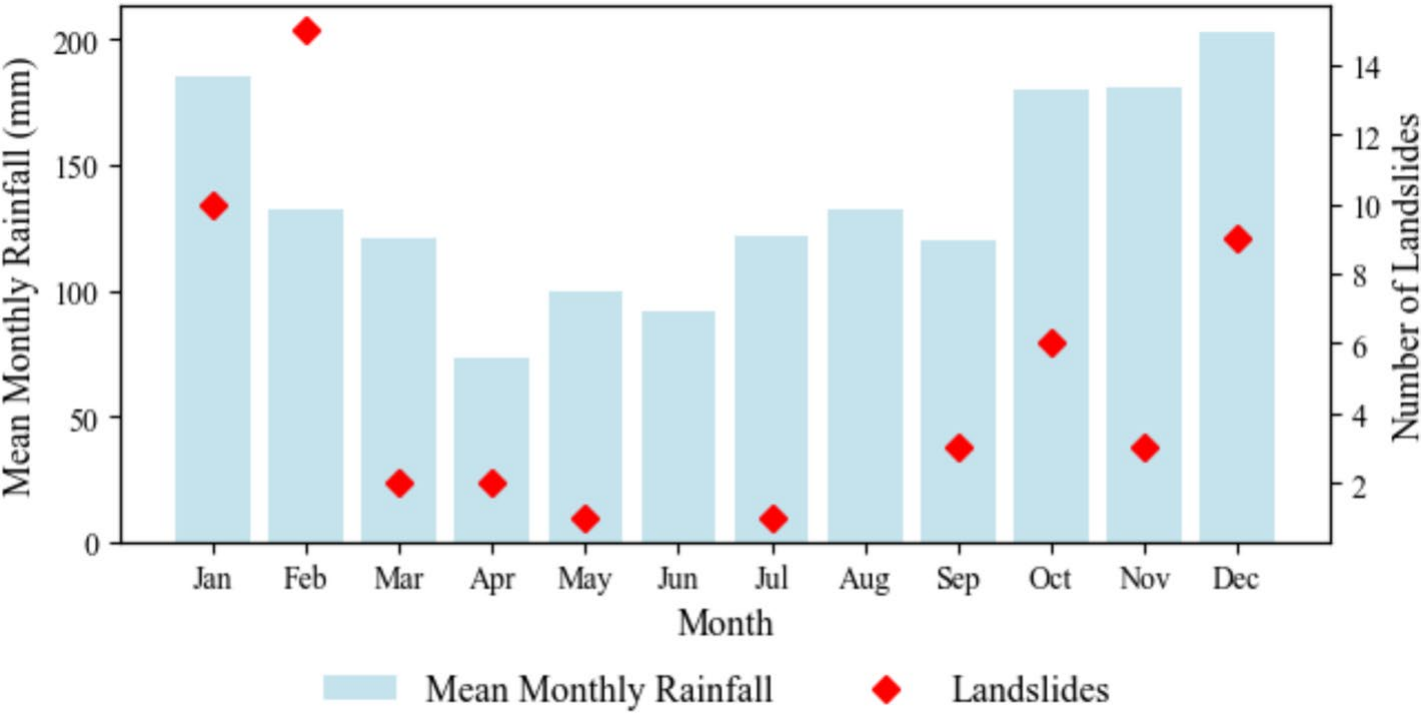
Average monthly rainfall (mm) and monthly distribution of landslides

This study utilised 15-min rainfall data spanning January 2005 to June 2023, collected from 52 automated rain gauges operated by Natural Resources Wales (NRW). The mean annual rainfall across the study area varies between 1000 and 2500 mm with 70% of the mean annual rainfall falling between September and March (see Fig. 2). According to the BGS landslide database, there are 58 landslides (Fig. 1) with known occurrence dates recorded over the period 2005–2023. Figure 2 shows that landslides most commonly occur during the wet autumn–winter season (September–February), emphasising the importance of rainfall as a primary driver.

According to the BGS Digital Soil Parent Material dataset (accessed via https://digimap.edina.ac.uk/geology), the study area exhibits geological heterogeneity, encompassing approximately 12 distinct major parent material types according to the European Soil Bureau (ESB) classification system. Overlaying these parent materials, the predominant soil types are sand, loam, sandy loam, clayey loam, and silty loam, which collectively account for about 70% of the total area. Recorded landslides primarily occurred within only four major parent material categories, namely, mudstone, sandstone, glacial till, and estuarine deposits. Correspondingly, the soils in these landslide-prone locations are mainly classified as loam to clayey loam or sandy loam to silty loam, reflecting moderate permeability.

## Methodology

**Fig. 3:**
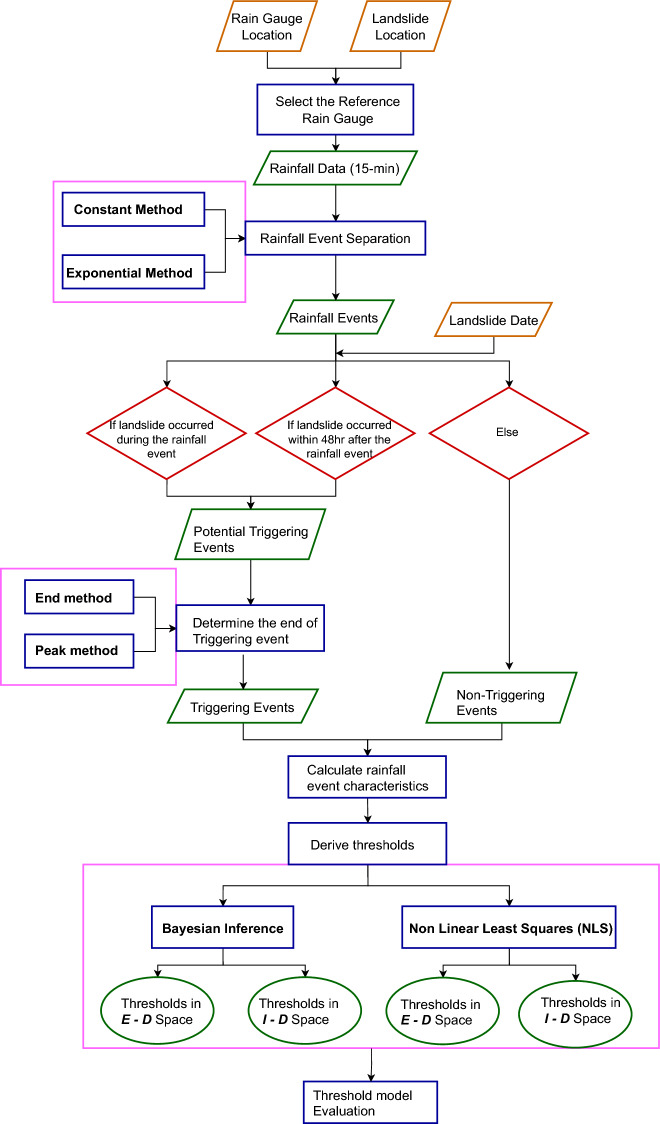
Flow chart describing the methodology used to generate and evaluate rainfall thresholds in this paper

This study follows a multi-step framework to evaluate the impact of rainfall characteristics on rainfall threshold estimation for landslide initiation. The main steps include: (i) rainfall event separation based on different MIT and triggering-event definitions, (ii) threshold derivation using BI and NLS methods, and (iii) threshold model performance evaluation. These steps are described in detail in the following sections, while Fig. 3 represents a flow chart summarising the overall methodology.

### Selection of reference rain gauge

The first step in determining the rainfall conditions responsible for landslide initiation is to select a rain gauge (herein known as the reference rain gauge) that closely represents rainfall conditions at the landslide location. Initially, all rain gauges within a 15 km circular buffer around each landslide location were identified as potential reference rain gauges. Note that rain gauges with more than 80% of data missing during the two years preceding the landslide were excluded. After this filtering, 56 landslides had more than one potential reference rain gauge while two landslides were removed from the database as they had no potential reference rain gauge. Each potential reference rain gauge was assigned weights (Table 1) based on four criteria: 1) proximity to the landslide-${W}_{D}$, 2) the absolute elevation difference between landslide location and a station-${W}_{E}$, 3) if the landslide location and gauge are situated within the same valley-${W}_{V}$, and 4) how comparable the rain gauge’s aspect is with that of the landslide-${W}_{A}$. All four parameters used in Eq. (1) were derived within a GIS environment (ArcGIS Pro) using a 50 m resolution Digital Terrain Model (DTM) acquired from the EDINA Digimap Ordnance Survey Collection (https://digimap.edina.ac.uk/os). The DTM was used to generate the Aspect layer and to delineate sub-basins, which were used to determine ${\mathrm{W}}_{\mathrm{A}}$ and ${\mathrm{W}}_{\mathrm{V}}$, respectively. Each criterion was rated as shown in Table 1, based on expert judgement, ensuring that gauges located closer in distance and elevation, within the same valley and with similar aspect to the landslide received the highest score. The rain gauge with the highest cumulative weight $\left( {W_{T} } \right)$ was selected as the reference rain gauge, considering all four criteria as equally influential as per Eq. (1).

1 $$ W_{T} = W_{D} + W_{E} + W_{V} + W_{A} $$

**Table 1:** Weight assignment for each criterion used for rain gauge selection

Criteria variable	Weight assignment
${\mathrm{W}}_{\mathrm{D}}$	Longest = 1, Shortest = no. of stations enclosed by the buffer
${\mathrm{W}}_{\mathrm{E}}$	Highest = 1, Lowest = no. of stations enclosed by the buffer
${\mathrm{W}}_{\mathrm{V}}$	Same valley = 2, Different valley = 1
${\mathrm{W}}_{\mathrm{A}}$	Same direction = 3, *Adjacent directions = 2, Any other = 1

^*^Based on an 8-part compass, “Adjacent directions” refers to the two neighbouring compass directions on either side of the aspect

### Separation of rainfall events

Different series of individual rainfall events can be extracted from a given raw rainfall data set, depending on the MIT criteria used to define a unique rainfall event. This choice of MIT is a major source of uncertainty in rainfall threshold modelling, and this study evaluates several MIT criteria set under two different approaches. For the first method, termed the “constant method”, MIT criteria were set at 4 h, 6 h, 12 h, 24 h, 48 h, 72 h, and 96 h and were not considered to vary throughout the year. These MIT criteria were chosen to include both the commonly applied criteria of 24 h, 48 h and 96 h (Sangelantoni et al. 2018; Leonarduzzi et al. 2021; Marino et al. 2020; Uwihirwe et al. 2020; Piciullo et al. 2017; Peruccacci et al. 2017; Melillo et al. 2015; Gariano et al. 2020; Zhao et al. 2020) and less explored sub-daily intervals (Tiranti and Rabuffetti 2010; Jiang et al. 2021), allowing for exploration of a wider range of temporal scales.

The second method, the “exponential method” (Iadanza et al. 2016; Wang et al. 2019), takes all the dry periods in the raw rainfall dataset into account to find a minimum dry period (MD) by iteratively estimating the coefficient of variation (CoV) of the dry periods and removing the smallest dry period until the CoV ($= \sigma /\mu )$= 1 (where the standard deviation ($\sigma$) and the mean $(\mu$) of dry periods are equal). The retained sample is assumed to consist only of the dry periods that separate independent rainfall events and fit into an exponential distribution described by $f\left(t\right)={\lambda e}^{-\lambda (t-MD)}$, where $t$ represents the duration of the dry period (h), ranging from MD to infinity, and $\lambda$ represents the parameter of the distribution which is equal to the inverse of the sample mean (Iadanza et al. 2016; Wang et al. 2019). Under the exponential method, a uniform average annual MIT value and separate monthly MIT values were identified.

### Distinguishing landslide triggering rainfall events

**Fig. 4:**
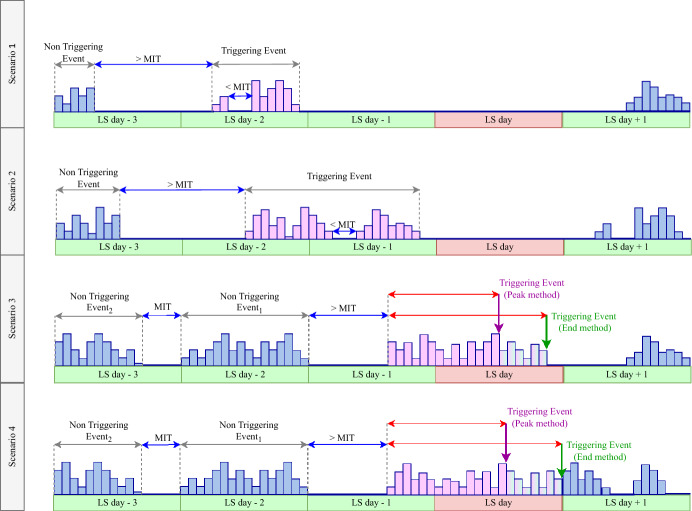
Illustration of four possible scenarios for identifying landslide triggering rainfall events and how rainfall records are separated into individual events based on MIT. In Scenarios 1 and 2, landslide occurs within 48 h following the end of the rain rainfall. In Scenario 3 and 4 are graphical explanation of the Peak and End method employed to determine the end of triggering event, when a landslide occurs during a rainfall event

In order to define rainfall thresholds for landslide initiation, rainfall events that trigger landslides need to be distinguished from those that do not. A rainfall event was considered a landslide triggering event if the failure occurred either during the rainfall event (Fig. 4 scenario 3 & 4) or within 48 h after the cessation of the rainfall event (Fig. 4 scenario 1 & 2) (Abraham et al. 2021; Melillo et al. 2018). In the latter case, the entire rainfall event is assumed to have contributed to the ground conditions leading to instability and can be considered a triggering event (Fig. 4). However, in the former scenario, any rainfall following failure is not relevant for threshold modelling (Melillo et al. 2015). Rarely, however, is the exact timing of landslide initiation recorded. To address this matter, two methods from the literature—the “End method” and the “Peak method”—were evaluated in this study (see Fig. 4 scenario 3 & 4). The End method considers the end of the rainfall event to be the last rainfall record on the day of the landslide, whereas in the Peak method the triggering rainfall was assumed to have coincided with the peak rainfall record on the day of the landslide.

### Estimation of rainfall thresholds

Rainfall trigger thresholds were estimated for both $I - D$ and $E - D$, using power-law relationship, as shown in Eq. (2).

2 $$R=\alpha {D}^{\beta }$$

where $\alpha$ and $\beta$ are model parameters, and $R$ represents $I$ or $E$ thresholds conditional on the duration $D$.

Three metrics: total event duration $D$ (hours), total event rainfall $E$ (mm) and mean event intensity $I$ (mm/h), were calculated for each triggering and non-triggering event. Given the limited number of local triggering events available in this study, the commonly used Ordinary Least Squares method was not adopted for model parameter estimation. Instead, two alternative approaches (1) Bayesian Inference and (2) Nonlinear Least Squares (NLS) were compared. These two methods have been suggested as more suitable options for cases with limited landslide data (Brunetti et al. 2010; Rossi et al. 2017); see Table 2 and will be presented in the following section.

**Table 2:** Mean and standard deviations of prior distributions of model parameters α and β

Threshold	μ_α__*prior*_	μ_β__*prior*_	σ_α__*prior*_	σ_β__*prior*_	References
E–D	8.6	0.43	0.9	0.04	Melillo et al. (2018)
I–D	9.4	− 0.56	2	0.1	Guzzetti et al. (2007)

^*^Standard deviation of *I* − *D* model parameters was assumed

#### Bayesian inference

Bayesian inference (*BI*) provides a probabilistic framework for updating existing knowledge of model parameters based on new data, while simultaneously quantifying the uncertainty associated with the updated parameters. Existing and updated knowledge of model parameters are expressed in terms of probabilities, known as “Prior” and “Posterior” probabilities, respectively. In this study, the Bayesian framework was used to update regional landslide threshold model fitting parameters with local landslide data to obtain a locally informed landslide threshold model relevant for the study area.

##### Prior specification

For numerical stability, the power-law relationship in Eq. (1) was linearised using a logarithmic transformation: $\log \left( X \right) = \log \left( \alpha \right) + \beta log\left( D \right)$. The prior distributions of $\mathit{log}\left(\alpha \right)$ and $\beta$ were assumed to follow a normal distribution, where ${\mu }_{log({\alpha }_{prior})}, {\mu }_{{\beta }_{prior}}$ are the means and ${\sigma }_{log({\alpha }_{prior})},{ \sigma }_{{\beta }_{prior}}$ are the standard deviations of the prior distributions of $\mathit{log}\left(\alpha \right)$ and $\beta$. The prior distributions were informed by work done (for regions in Italy) by Guzzetti et al. (2007) for $I - D$ thresholds and Melillo et al. (2018) for $E-D$ thresholds; see Table 2. ${\mu }_{log({\alpha }_{prior})}$ was obtained directly by log transforming ${\mu }_{log({\alpha }_{prior})}$ while ${\sigma }_{log({\alpha }_{prior})}$ was approximated as ${\sigma }_{{\alpha }_{prior}}/{\mu }_{{\alpha }_{prior}}$.

##### Likelihood function

For a given set of observed data points (${D}_{i}, {R}_{i}, {y}_{i}$), where *i* refers to an individual rainfall event, ${R}_{i}$ represents either ${I}_{i} or {E}_{i}$ and ${y}_{i}$ is a binary indicator that signifies landslide occurrence (${y}_{i}=1$ if landslide occurs, and ${y}_{i}=0$ otherwise), the logarithm of predictions (${R}_{i,pred}$) at each ${D}_{i}$ were obtained using the prior parameter; see Eq. (3).

3 $$\mathit{log} \left({R}_{i, pred}\right) =\mathit{log}\left({\alpha }_{prior}\right)+{\beta }_{prior}\mathit{log}\left(D\right)$$

The difference between the predicted and observed values, $\mathit{log}\left({R}_{i,pred}\right)-\mathit{log}({R}_{i})$, is passed to a logistic function (see Eq. (4)), which maps this difference to a probability between [0,1]. Larger negative differences (where the observed value exceeds the predicted threshold in log-space) correspond to a higher landslide probability, whereas larger positive differences correspond to a lower probability.

4 $$ p_{i} = 1/\left( {1 + {\mathrm{exp}}\left( { - \left( {\log \left( {\alpha_{prior} } \right) + \beta_{prior} \log \left( D \right) - {\mathrm{log}}\left( {R_{i} } \right)} \right)} \right)} \right) $$

For each observed rainfall event $i$, the landslide occurrence indicator ${y}_{i}$ follows a Bernoulli distribution with probability ${p}_{i}$. Therefore, the likelihood of observing ${y}_{i}$ given $\mathrm{log}\left({\alpha }_{prior}\right)$ and ${\beta }_{prior}$—can be expressed as (see Eq. (5)):

5 $$P\left({y}_{i}|\mathrm{log}\left({\alpha }_{prior}\right),{\beta }_{prior}\right)={p}_{i}^{{y}_{i}}{(1-{p}_{i})}^{(1-{y}_{i})}$$

Based on the likelihood function and prior probabilities, approximations for posterior probabilities (see Eq. (6)) were generated by MCMC methods using the PyMC3 Python library.

6 $$ P\left( {\log \left( {\alpha_{posterior} } \right),\beta_{posterior} {|}y} \right)\alpha P\left( {y{|}\log \left( {\alpha_{prior} } \right),\beta_{prior} } \right) \times P\left( {\log \left( {\alpha_{prior} } \right)} \right) \times P\left( {\beta_{prior} } \right) $$

The updated threshold model was derived from the median values of the posterior distributions for $\alpha$ and $\beta$ samples. Additionally, threshold equations were generated for each accepted posterior sample, and evaluating these equations across all samples provided the 95% credible intervals for the updated threshold.

#### Nonlinear least squares

Nonlinear Least Squares (NLS) is a common method used to estimate model fitting parameters in nonlinear regression models. A nonlinear model is typically expressed as in Eq. (7) with a nonlinear function $f$ and an error term $\varepsilon$ as:

7 $$Y=f\left(D, \alpha ,\beta \right)+\varepsilon $$

where $Y$ is the observed response (in this study either $I$ or $E$), $f\left(D, \alpha ,\beta \right)$ is the nonlinear function of the predictor variable $D$ with $\alpha$ and $\beta$ parameters and $\epsilon$ is the error term. The objective of NLS is to find the parameter values that minimise the sum of squared residuals ($S$) (See Eq. (8)) (Ritz and Streibig 2008):

8 $$S=\sum_{i=1}^{n}{\left[{Y}_{i}-f\left(D, \alpha ,\beta \right)\right]}^{2}$$

In contrast to linear regression, where analytical solutions are readily available, no closed-form solution exists for estimating parameters (Ritz and Streibig 2008) in nonlinear regression due to the nonlinearity of $f\left(D, \alpha ,\beta \right)$. Consequently, iterative numerical optimization algorithms such as Gauss–Newton or Levenberg–Marquardt, are required to minimise $S$ (Ritz and Streibig 2008). Selecting an appropriate nonlinear function and initial parameter values is critical for achieving a reliable fit (Ritz and Streibig 2008).

The power-law function in Eq. (2) was selected as the nonlinear function that describes triggering events, and the Levenberg–Marquardt algorithmwas used to minimise $S$. Initial parameter values (Table 2) were taken from regional studies. A bootstrap approach with 1000 iterations was used to resample and refit the model in order to quantify the uncertainty in the approach and establish confidence intervals. The resulting best fit describes the 50% non-exceedance threshold (${\alpha }_{50},{\beta }_{50})$.

Similar to Rossi et al. (2017), this study adopts a 5% non-exceedance threshold as the critical landslide initiation threshold. It was assumed that the 5% non-exceedance threshold is parallel to the fitted model (Brunetti et al. 2010), therefore slope parameter $\beta$ does not change (${\beta }_{5}= {\beta }_{50})$. The $\alpha$ value for the 5% non-exceedance threshold, ${\alpha }_{5}$, was estimated using Eq. (9):

9 $${\alpha }_{5}= {\alpha }_{50}-\delta $$

where $\delta$ denotes the 5th percentile of the probability density function of the residuals, computed in log space as $\mathit{log}\left({Y}_{predicted}\right)- \mathit{log}\left({Y}_{observed}\right)$.

### Threshold model evaluation

The performance of threshold models was evaluated in terms of confusion matrix-based metrics, i.e., False Positive Rate (FPR), True Positive Rate (TPR), False Negative Rate (FNR) and True Skill Statistic (TSS). A confusion matrix is used to determine the number of correctly and incorrectly classified events. In the context of landslide threshold modelling:

- A True Positive (TP) occurs when a triggering event is correctly classified above the threshold.
- A True Negative (TN) occurs when a non-triggering event is correctly classified below the threshold.
- A False Positive (FP) is a non-triggering event misclassified above the threshold.
- A False Negative (FN) is a triggering event misclassified below the threshold.

FPR measures how many actual negatives are incorrectly predicted as positives and defined as $FPR = FP/(FP+TN)$, while $TPR = TP/(TP+FN)$ is the fraction of correctly predicted triggering events, and $FNR = FN/(TP+FN)$ is the fraction of missed triggering events. The TSS balances TPR and FPR as described by $TSS = TPR {-} FPR$.

Despite the possibility of misleading outcomes due to highly imbalanced datasets, the Receiver Operating Characteristics (ROC) approach was also employed, where the Area Under the ROC curve (ROC-AUC) evaluates the discriminative power of a threshold model.

## Results and discussion

### Effects of MIT on rainfall event characteristics

To determine the impact of varying MIT on rainfall event separation, the average annual number of rainfall events was calculated at each station for a range of MIT values. Table 3 tabulates the mean and standard deviation of the average annual number of rainfall events under each MIT scenario. There is a distinctive decreasing trend in the number of annual rainfall events as MIT increases. This behaviour, which is consistent with findings from other geographical contexts by Wang et al. (2019) and Tu et al. (2023) in China, Chin et al. (2016) in Malaysia, and Molina-Sanchis et al. (2016) in Spain among others, is expected, as some individual rainfall events separated by a small MIT would be subsumed into one larger rainfall event as MITs increase. Furthermore, a decline in the standard deviation with increasing MIT suggests a reduction in station-to-station variation in the number of annual rainfall events, reflecting a greater consistency in rainfall patterns across the study area when aggregated over a longer duration.

**Table 3:** Mean and standard deviation of the annual rainfall events (average number of events per annum) computed across all stations for different MIT criteria using: (a) the Constant method, and (b) the Exponential method

	Constant method							Exponential method
	4 h	6 h	12 h	24 h	48 h	72 h	96 h	
Mean	134	118	87	53	30	22	18	65
Standard deviation	10	7	3	2	2	1	1	3

**Fig. 5:**
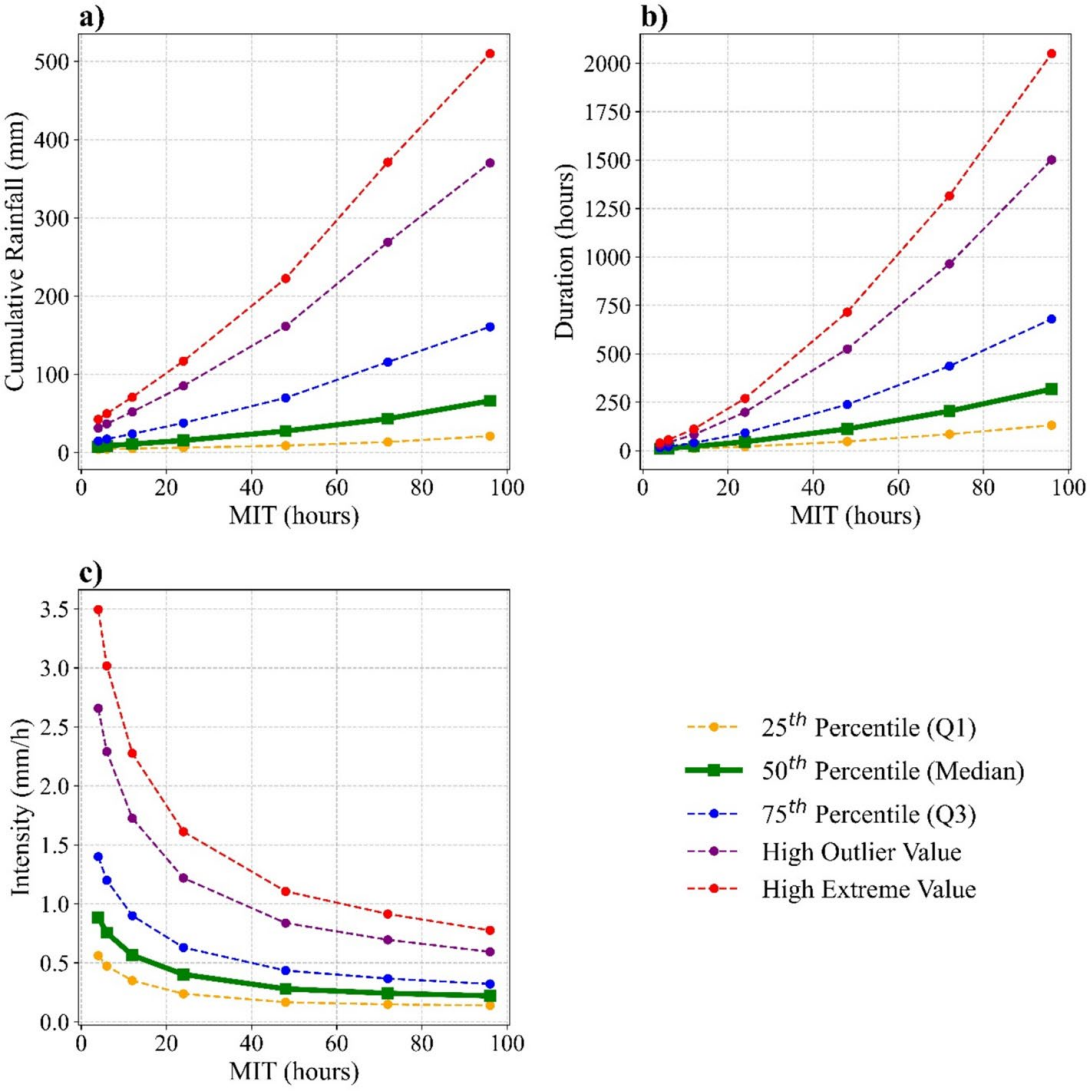
Variation of descriptive statistics describing rainfall event characteristics with increasing MIT (constant method); **a** Cumulative event rainfall, **b** Total event duration, and **c** Mean event intensity. High outlier value (HOV) = Q1 + 1.5(Q3–Q1). High extreme value (HEV)

Figure 5a–c shows the average rainfall depth, duration and average intensity of events plotted against MIT. The plots highlight that as MIT increases, total rainfall (a) and rainfall duration (b) increase, while rainfall intensity (c) decreases. The mean event duration and mean event rainfall at MIT = 48 h are about 1476% longer and 409% larger than those at MIT = 4 h, while the mean event intensity over the same period decreases by 68.29%. These trends indicate that event characteristics are strongly influenced by MIT variation, with event duration exhibiting the highest sensitivity to increasing MIT. Although similar trends were reported by Tu et al. (2023) in China, the sensitivity of intensity to increasing MIT in South Wales is noticeably lower. This may reflect the predominance of prolonged, low-intensity rainfall in the South Wales region. However, it highlights the regional variability of rainfall patterns, while underscoring the importance of region-specific thresholds, and MIT choice in rainfall characterisation.

**Fig. 6:**
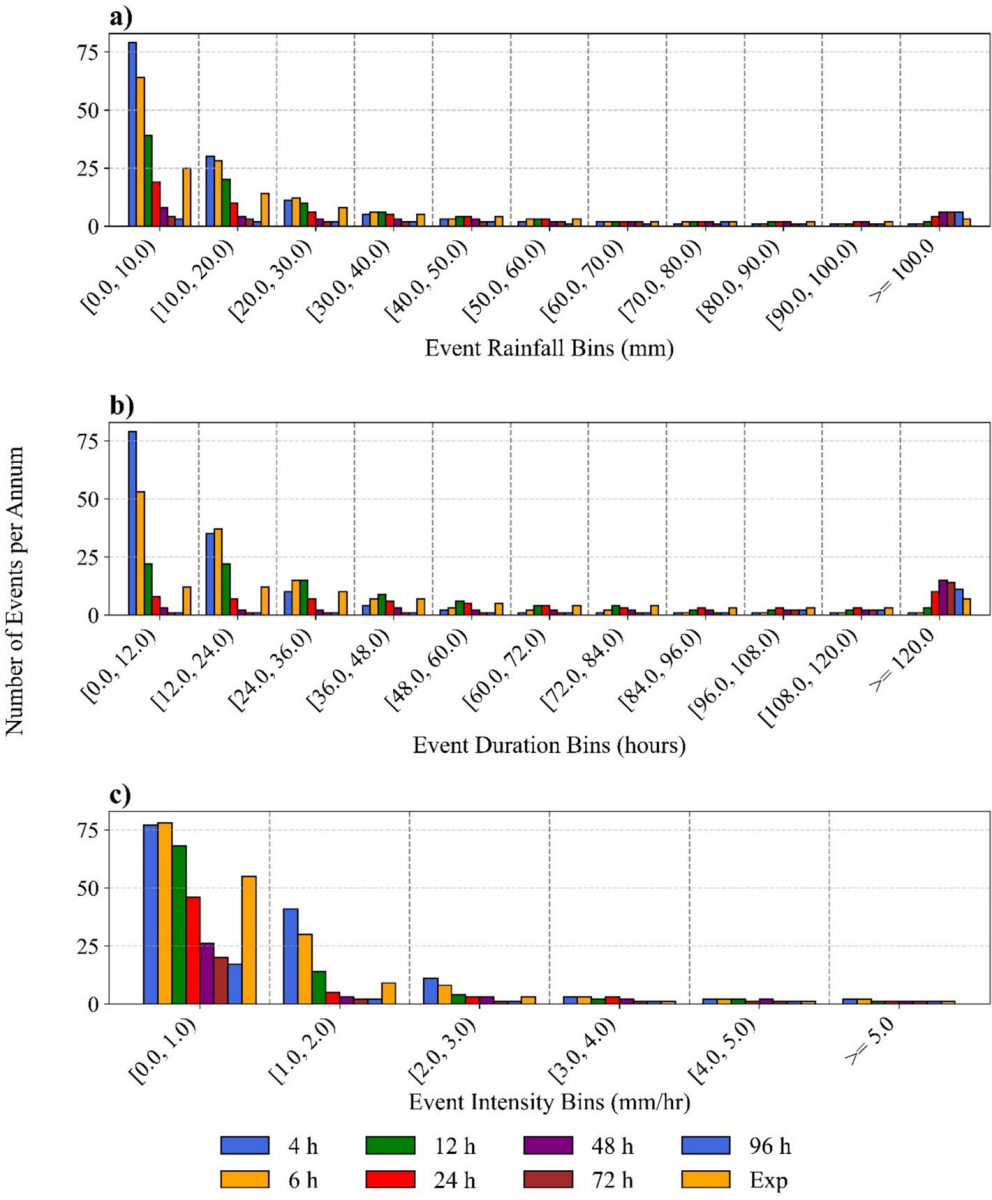
Variation of the mean annual number of rainfall events in each bin under all MIT criteria; **a** Event rainfall, **b** Event duration, and **c** Event intensity. Numerical values are MIT considered under the constant method. Exp refers to the exponential method

Across all MITs, rainfall events with total rainfall below 20 mm were the most prevalent in the study area (Fig. 6a), with the number of events in the lower rainfall bins decreasing as MIT increases. For shorter MITs (4 h and 6 h), most rainfall events in the study area have durations below 24 h, with over 70% of mean annual events falling within this range (Fig. 6b). In contrast, as MIT increases to 24 h and 96 h, the proportion of long-duration events ($\ge$ 5 days) rises significantly, reaching 17%, 41%, 54% and 48% for MITs of 24 h, 48 h, 72 h, and 96 h, respectively. Rainfall events in the study area are predominantly low to moderate in intensity, with over 50% of events exhibiting intensities below 1 mm/h across all MITs (Fig. 6c). However, occasional extreme rainfall events with significantly higher intensities suggest that the study area experiences rare but intense rainstorms. These patterns in total rainfall and duration mirror the trends observed in other regions—for instance, Tu et al. (2023) reported that the frequency of < 20 mm events declined, while those > 50 mm increased with increasing MIT, while Chin et al. (2016) observed that in Malaysia, the proportion of rainfall events exceeding 24 h in duration was negligible when MIT was below 10 h but exceeded 20% when MITs were above 22 h.

According to Fig. 6b, a steady decline in the frequency of rainfall events across duration bins is observed for all MITs except for MITs of 48 h, 72 h and 96 h, which exhibit a much flatter distribution across durations except in the > 120 h bin. These trends in event duration, coupled with the substantial 87% reduction in the mean of annual rainfall events (Table 3) and the corresponding decrease in standard deviation as MIT increases from 4 to 96 h, suggest that rainfall in the study area tends to occur in multiple bursts over an extended duration, with brief dry intervals in between, resulting in a substantially higher number of independent rainfall events at lower MITs. However, the impact of MIT on landslide threshold modelling cannot solely be explained by the dominant rainfall patterns, as rainfall induced stability is governed by the amount of water that infiltrates a slope and the water balance within the slope (Greco et al. 2023). In highly permeable soils, rainwater can infiltrate and percolate to depth quickly, causing substantial changes in saturation over short durations. On the other hand, in soils with low permeability, the near-surface soil might remain saturated from a previous rainfall event, and hence two successive bursts separated by a shorter dry period cannot be considered as two independent events. Therefore, for rainfall induced landslide thresholds, reconstruction of rainfall events by a shorter MIT could be better suited to highly permeable soils, and a longer MIT may be better suited to low permeability soils.

#### Exponential method

**Fig. 7:**
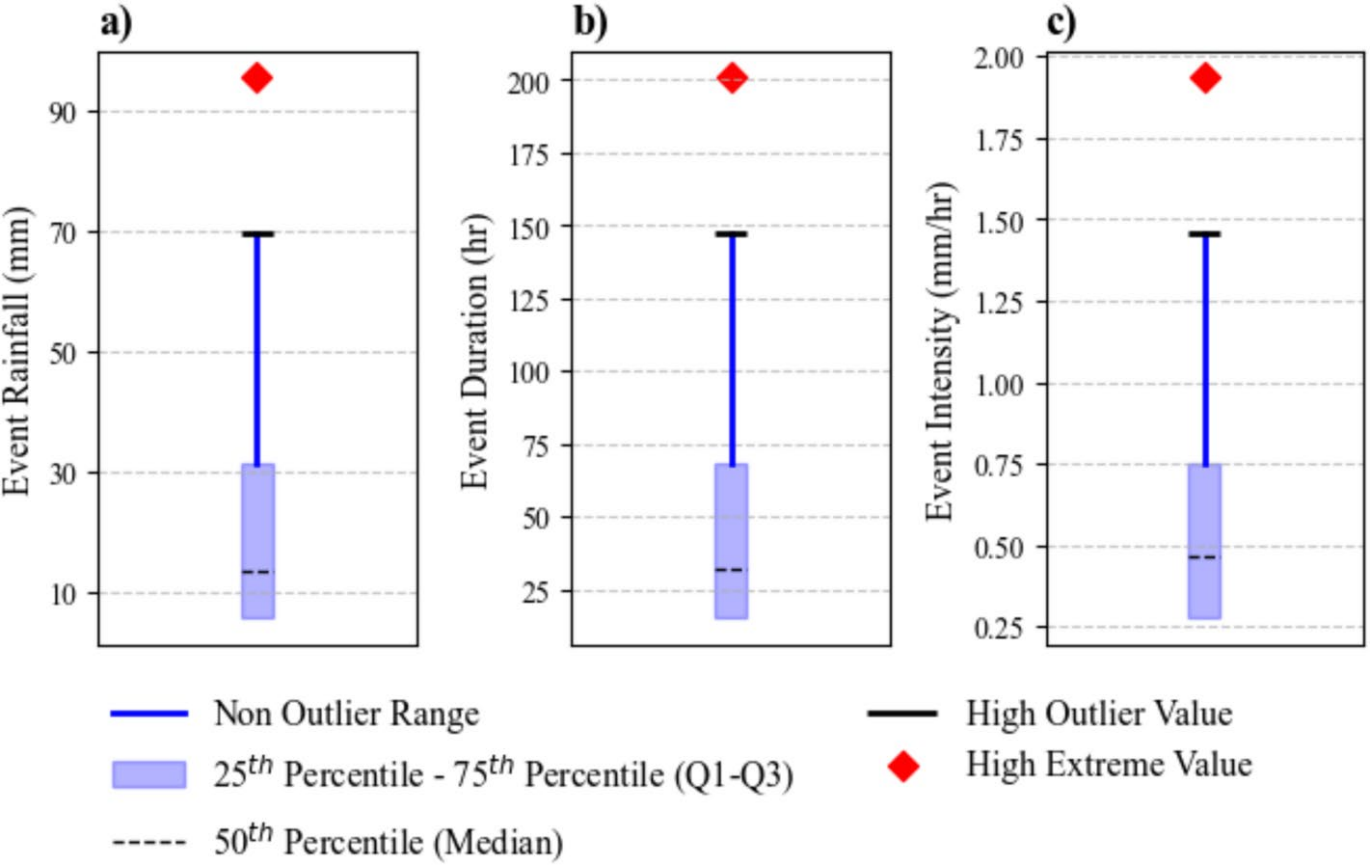
Box pots of rainfall event characteristics under exponential method; **a** Event rainfall, **b** Event duration, and **c** Event intensity. High outlier value (HOV) = Q1 + 1.5 (Q3–Q1). High extreme value (HEV) = Q1 + 2.5(Q3–Q1). Metric values between HOV and HEV are outlier, and metric values greater then HEV are extremes

**Fig. 8:**
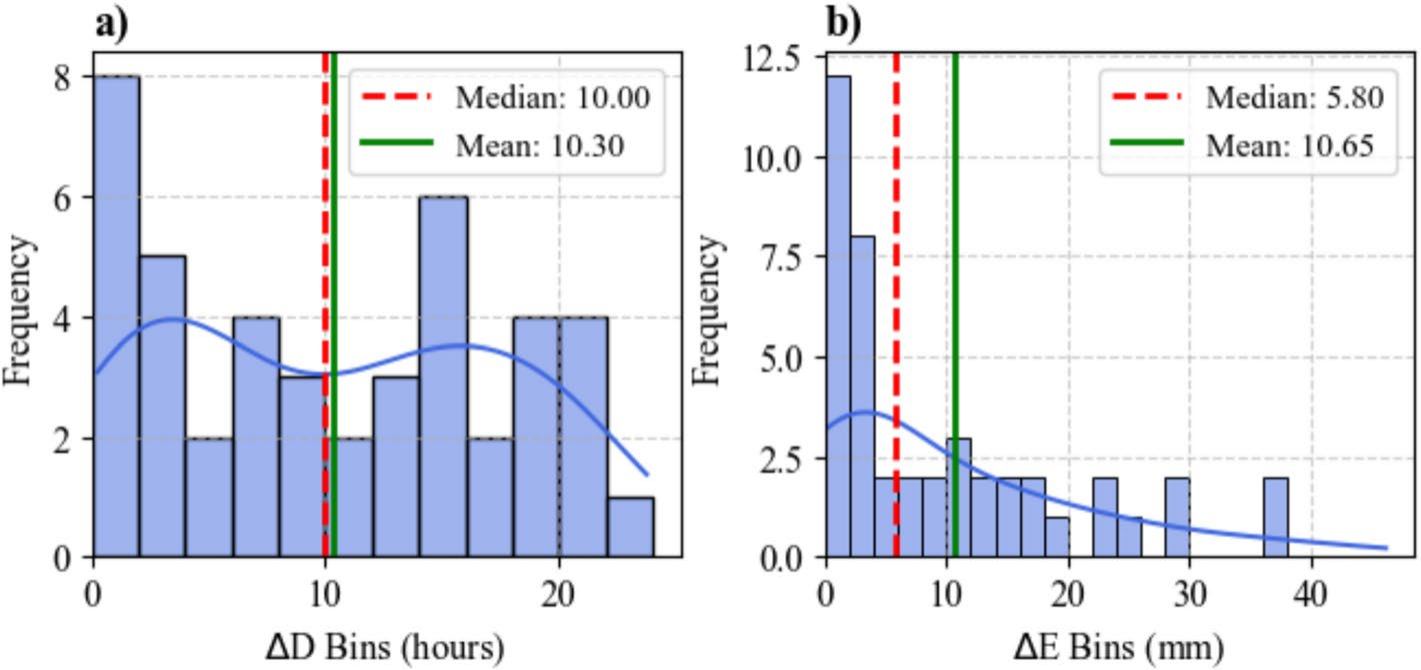
Histogram of differences in **a** event duration (∆D) and **b** event rainfall (∆E) between the End and Peak methods. Bin size = 2 in respective units

**Fig. 9:**
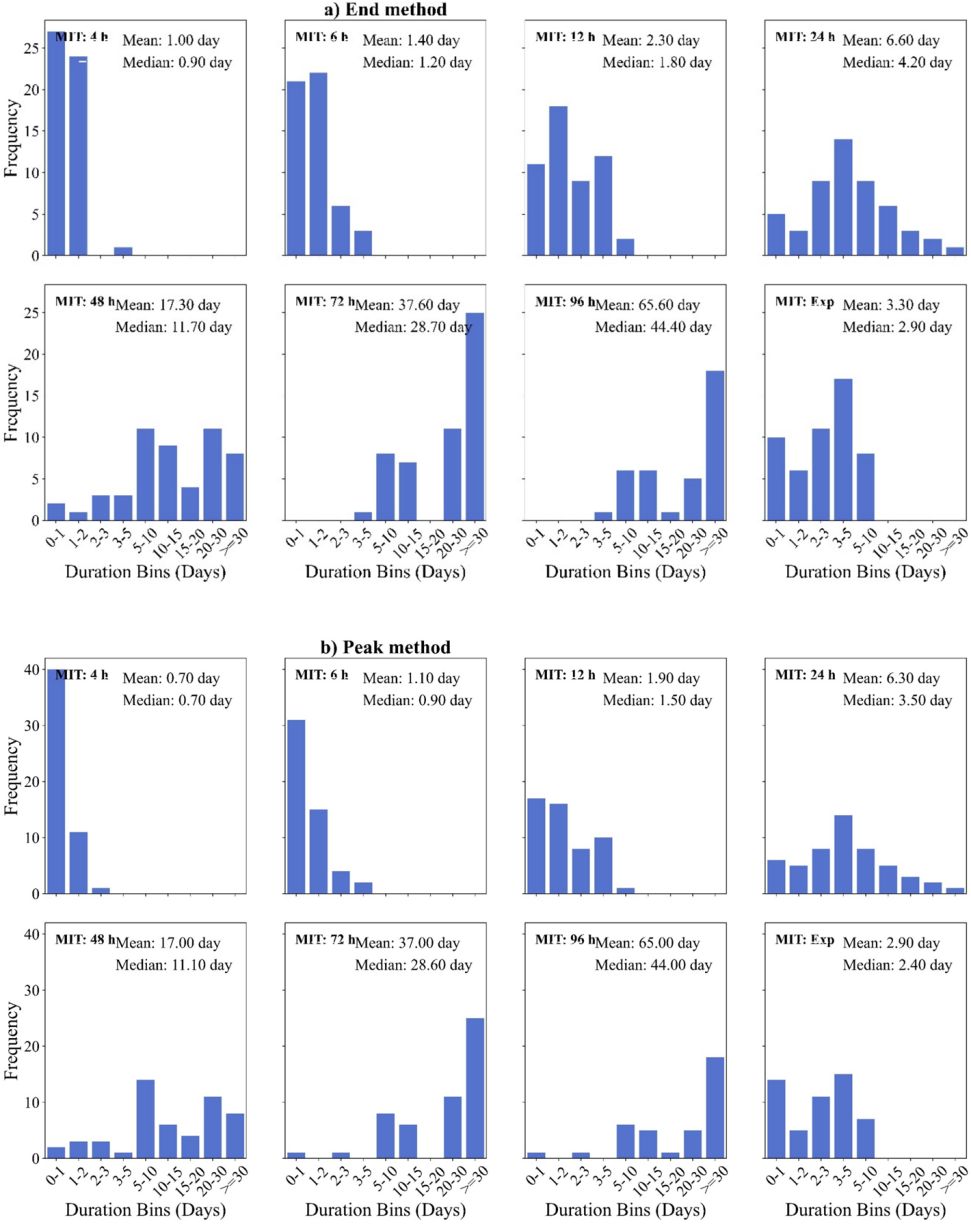
Histograms illustrating the distribution of triggering event durations identified using the **a** End method and **b** Peak method across each MIT criteria. Bin sizes are unequal, respective mean and median values are indicated within each plot

**Fig. 10:**
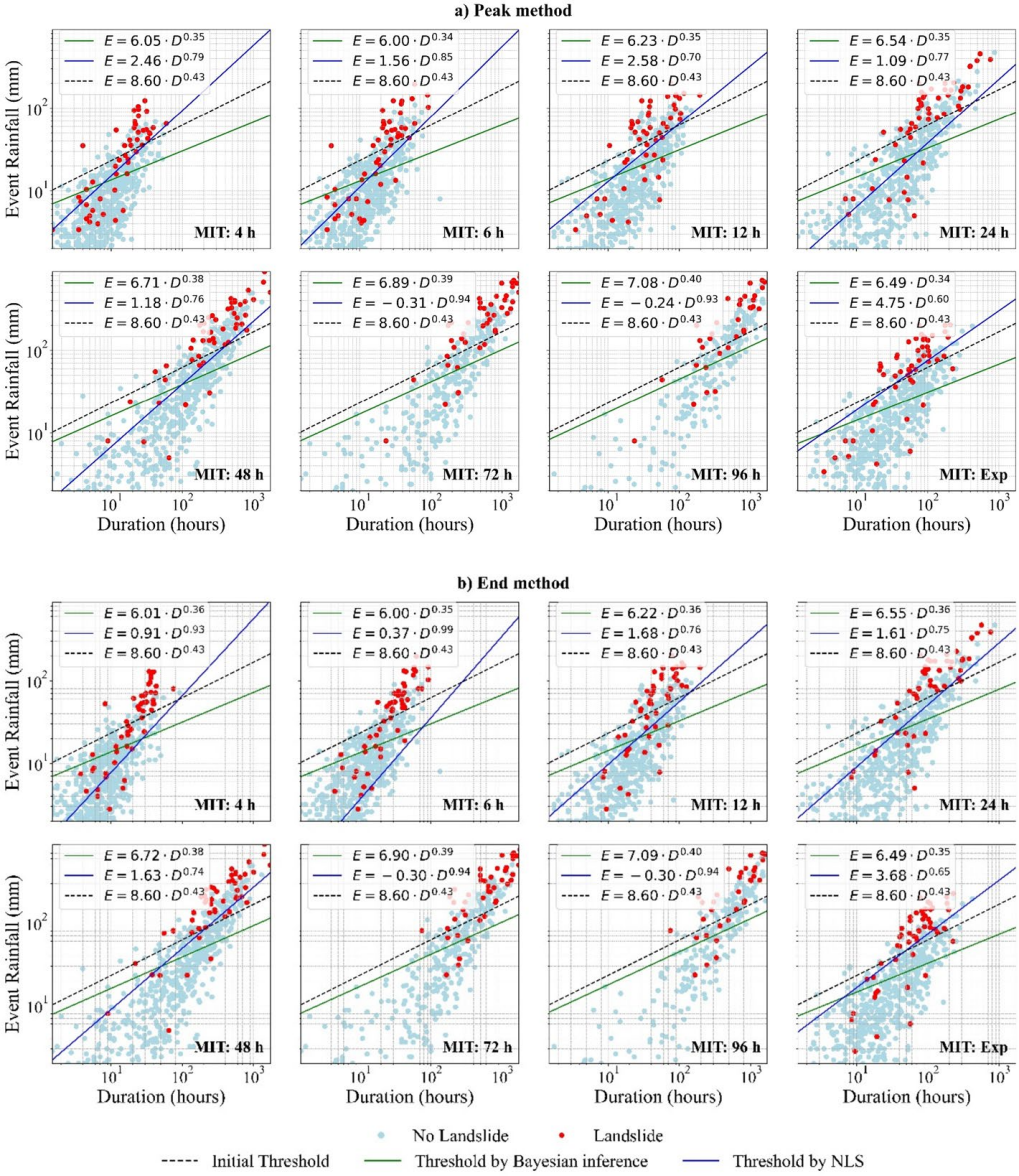
E–D thresholds estimated using Bayesian Inference and NLS under different MIT criteria, compared to the initial threshold model by Melillo et al. (2018). **a** Thresholds derived from triggering events identified by using the Peak method and **b** Thresholds derived from triggering events identified by using the End method

**Fig. 11:**
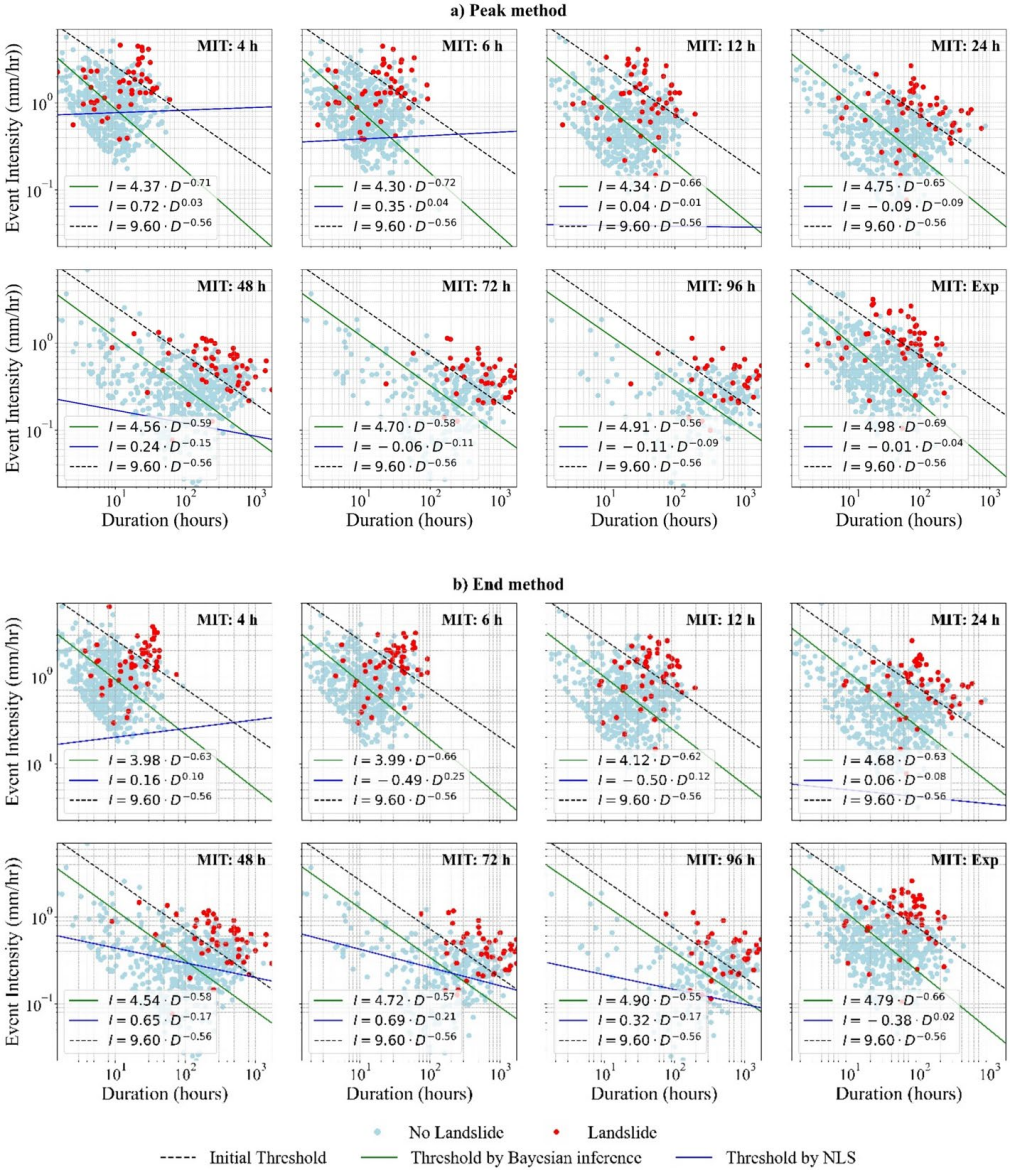
I–D thresholds estimated using Bayesian Inference and $\mathrm{NLS}$ for different MIT criteria, compared to the threshold model by Guzzeti et al. 2007. **a** Thresholds derived from triggering events identified using the Peak method and **b** Thresholds derived from triggering events identified using the End method

**Fig. 12:**
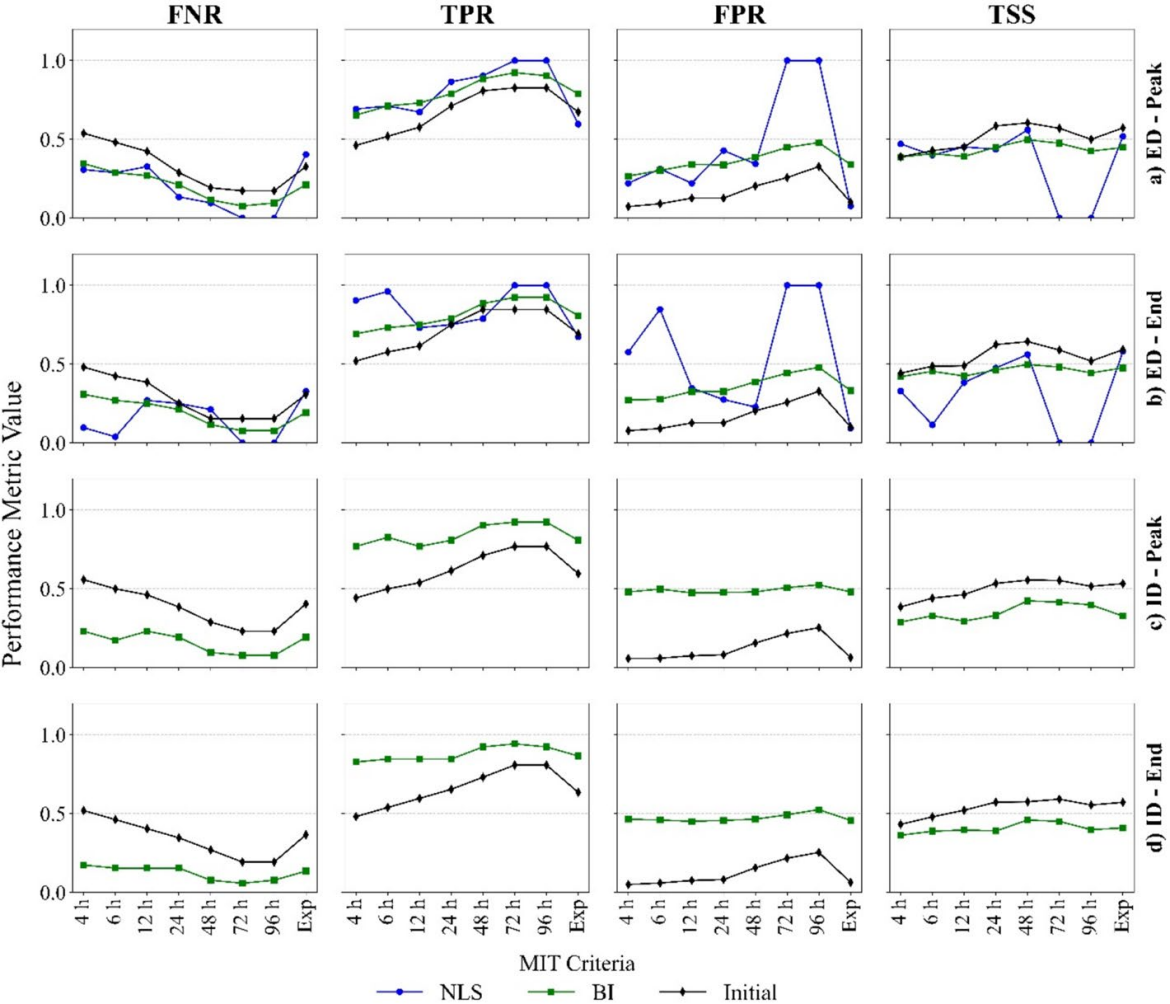
Variation of confusion matrix-based evaluation measures with MIT criteria. **a** E-D thresholds under Peak method, **b** E-D thresholds under End method, **c** I-D thresholds under Peak method and **d** I-D thresholds under End method

Using the exponential method, both a single MIT value for the whole year as well as MIT values for each month of the year were calculated. Monthly MIT values are given in Table 4. The yearly MIT was 25 h. Since MIT = 24 h was already tested under constant method, only the monthly MIT values were used to obtain the results (in Tables 3, 4, 5 and 6 and Figs. 6, 7, 8, 9, 10, 11 and 12) associated with the exponential method. Results from this method revealed considerable monthly variability in MIT. In the study region, warmer months generally exhibited longer dry intervals than colder months. Iadanza et al., (2016), who also confirmed seasonal variation in MIT, observed the opposite pattern in Italy, where dry periods in winter months were longer compared to summer months. This contrast highlights the region-specific nature of seasonal rainfall patterns and reinforces the importance of region-specific MIT values.

**Table 4:** MIT values for each month obtained by exponential method

	Jan	Feb	Mar	Apr	May	Jun
MIT (h)	11	19	24	26	42	18

	Jul	Aug	Sep	Oct	Nov	Dec
MIT (h)	27	17	23	17	11	18

**Table 5:** Parameter estimates and their confidence intervals for E–D thresholds under each method, along with model performance evaluation results based on ROC-AUC

MIT	Peak Method					End method				
	α	β	CI_α_	CI_β_	ROC-AUC	α	β	CI_α_	CI_β_	ROC-AUC
(a) *BI*										
4 h	6.05	0.35	2.20	0.14	0.694	6.01	0.36	2.23	0.14	0.710
6 h	6.00	0.34	2.23	0.14	0.705	6.00	0.35	2.24	0.14	0.727
12 h	6.23	0.35	2.33	0.13	0.695	6.22	0.36	2.29	0.13	0.712
24 h	6.54	0.35	2.47	0.12	0.725	6.55	0.36	2.46	0.12	0.731
48 h	6.71	0.38	2.53	0.11	0.749	6.72	0.38	2.57	0.11	0.749
72 h	6.89	0.39	2.66	0.17	0.737	6.90	0.39	2.69	0.17	0.741
96 h	7.08	0.40	2.71	0.16	0.712	7.09	0.40	2.76	0.16	0.722
Exp	6.49	0.34	2.41	0.13	0.724	6.49	0.35	2.42	0.13	0.738
(b) *NLS*										
4 h	2.46	0.79	5.70	0.61	0.735	0.91	0.93	4.70	0.95	0.664
6 h	1.56	0.85	4.63	0.58	0.700	0.37	0.99	2.70	0.50	0.557
12 h	2.58	0.70	6.88	0.43	0.726	1.68	0.76	5.67	0.45	0.691
24 h	1.09	0.77	5.25	0.35	0.718	1.61	0.75	5.28	0.34	0.738
48 h	1.18	0.76	6.68	0.46	0.780	1.63	0.74	7.25	0.44	0.781
72 h	− 0.31	0.94	1.73	0.39	0.500	− 0.30	0.94	1.76	0.39	0.500
96 h	− 0.24	0.93	1.56	0.36	0.500	− 0.30	0.94	1.54	0.37	0.500
Exp	4.75	0.60	12.53	0.42	0.760	3.68	0.65	11.48	0.42	0.790

The mean of the annual rainfall events identified using the exponential method falls between those obtained using the constant method for MIT = 12 h and MIT = 24 h (Table 3). When Fig. 7 is compared with Fig. 5, it is evident that, for each metric, the results of the exponential method consistently fall between those of MIT = 12 h and MIT = 24 h. This consistency across all observations suggests that the exponential method may provide a robust alternative for event separation by avoiding both excessive fragmentation and aggregation of rainfall events.

### Comparison of triggering event characteristics identified by different methods

To compare how often the End and Peak methods identify different rainfall events as triggering events, differences in event duration ($\Delta D)$, total event rainfall $(\Delta E$) and event intensity ($\Delta I)$ were computed for each landslide. The differences are defined as Eq. (10):

10 $${\Delta X}_{ m,i}={\left({X}_{E}\right)}_{m,i}-{\left({X}_{P}\right)}_{m,i}$$

where $X$ represents $D$, $E$, or $I$ and ${\left({X}_{E}\right)}_{m,i}$ and ${\left({X}_{P}\right)}_{m,i}$ are the values derived from the end and peak methods, respectively. The subscripts $m$ and $i$ specify the MIT criterion and the landslide event.

In 85% of the landslides, $\Delta D, \Delta E,$ and $\Delta I$ are non-zero, indicating that the two methods usually select different triggering events. As expected, $\Delta D\ge 0$ and $\Delta E\ge 0$ for all cases, because the End method extends rainfall beyond its peak. The outcome $\Delta D=0$ occurs when the peak rainfall of the landslide day is also the last record of that day or if negligible rainfall (< 1 mm) is reported on the landslide day. In such cases, the event terminates no more than 48 h before the landslide, causing both methods to converge to the same event.

When both $\Delta D$ and $\Delta E$ are non-zero, $\Delta I$ can be positive or negative. A negative $\Delta I$ (i.e. lower intensity under the end method) occurs when the additional post-peak rainfall is relatively small compared to the increase in duration imposed by the End method.

Histograms of non-zero $\Delta D$ (Fig. 8a) show that differences in event duration range from near zero (indicating almost identical durations) to approximately 24 h, where the End method substantially extends rainfall events. In contrast $\Delta E$ (Fig. 8b) is positively skewed with most values clustered below 4 mm. The median values of $\Delta D$ (10 h) and $\Delta E$ (5.8 mm) support a negative $\Delta I$, indicating that while the end method captures longer durations, the extra post-peak rainfall is generally not large enough to maintain peak intensities. However, the long tail of $\Delta E$ (up to 40 mm) suggests that some landslide triggering events may receive substantial additional rainfall after the peak ($+\Delta I$), which is only captured by the End method.

### Effects of MIT on capturing antecedent conditions

For an MIT of 4 h, nearly all triggering events last less than 48 h (2 days), with mean durations of 24 h (End) and 17 h (Peak), see Fig. 9. This suggests that MIT = 4 h captures short-term (< 24 h) antecedent rainfall. As MIT increases, event durations gradually extend, indicating that larger MIT values provide an improved representation of antecedent conditions. A more pronounced shift occurs at MIT = 24 h, where the mean event duration increases to $\sim$ 6 days (median $\sim 3.5$ days), with most antecedent durations falling within 3–10 days. At MITs of 48 h, 72 h and 96 h, nearly 50% of triggering events last longer than10 days, implying that longer MITs incorporate cumulative rainfall beyond individual storm events, capturing the prolonged moisture accumulation critical for landslide initiation.

Consequently, sub-daily MIT values can underestimate the duration of triggering events, particularly for slopes where antecedent moisture plays a key role. In contrast, longer MITs (24–96 h) provide a more comprehensive view of antecedent rainfall. The exponential method closely aligns with MIT = 12 h but produces slightly longer durations of 3.3 days for End method and 2.9 days for Peak method. This suggests that the exponential method may be more effective than the manual sub-daily MIT Criteria in representing antecedent conditions.

### Rainfall thresholds

In this section, the importance and influence of MIT definition on the rainfall thresholds is investigated, using Bayesian inference and NLS as outlined in Sect. 3.4..

#### Comparison of existing thresholds with BI and NLS methods

In $E-D$ space, the slope parameters, $\beta$, estimated by *BI* are only slightly lower than the initial $\beta$ from Melillo et al. (2018) (see Table 2), while the values of $\alpha$ are consistently and noticeably smaller across all MITs for both Peak and End methods (Table 5). This means that *BI* maintains the overall shape of the initial $E - D$ threshold while refining its position (changing $\alpha$) based on the available data (Fig. 10). While yielding unrealistic thresholds (negative $\alpha$) at MITs of 72 h and 96 h, the *NLS* approach yielded significantly lower $\alpha$ and higher $\beta$ (Table 5) at all other MIT values compared to both the initial (Table 2) and *BI*-derived $E-D$ threshold parameters (Table 5), resulting in steeper curves that are highly sensitive to event duration. Model parameter estimates reveal that *BI*-derived thresholds predict landslides at lower cumulative rainfall (total event rainfall) for any given duration compared to the initial $E-D$ threshold. *NLS*-derived thresholds predict even lower rainfall requirements for landslide initiation, except for longer durations (around the 75th percentile), where they exceed the original $E-D$ threshold. Therefore, only at shorter durations do the NLS results align with Peres and Cancelliere (2021), who compared various model parameter estimation methods and concluded that relying only on triggering events leads to underestimated thresholds. These results indicate that landslides in South Wales may be triggered by substantially lower rainfall amount compared to regions like Italy (e.g.: Melillo et al. 2018). Interestingly, the empirical $E-D$ threshold (5% non-exceedance) derived by Abraham et al. (2021) for a region in India is of similar magnitude to the *BI*-derived thresholds for South Wales when larger MIT values are applied. This comparison highlights how empirical thresholds vary significantly across geographic settings.

In the $I-D$ space, *BI* produced thresholds with lower α values than the initial $I-D$ model (see Table 2) across all MITs for both Peak and End methods (Table 6). However, the $\beta$ parameters of the $I-D$ thresholds were consistently higher than those from Guzzetti's (2007) $I-D$ model, except for MIT = 48 h, 72 h, and 96 h, where the difference was minimal. This outcome indicates that the rate at which required intensity decreases with duration became steeper in the updated $I-D$ thresholds. These findings also imply that the intensity required to trigger landslides in South Wales is generally lower than that suggested by the initial $I-D$ threshold by Guzzetti et al. (2007) for Italy. Martinovic et al. (2018) also demonstrated that landslides in Ireland, a region with a temperate maritime climate broadly comparable to South Wales, can be triggered by rainfall intensities lower than those observed in Italy by Guzzetti et al. (2007). However, the $I-D$ thresholds identified in this study area are significantly lower than those reported for natural slopes in Ireland by Martinovic et al. (2018) but are closer to those derived for man-made slopes.

**Table 6:** Parameter estimates with confidence intervals for I–D thresholds for each method, along with model evaluation results based on ROC-AUC

MIT	Peak method					End method				
	α	β	CI_α_	CI_β_	ROC-AUC	α	β	CI_α_	CI_β_	ROC-AUC
(a) *BI*										
4 h	4.37	− 0.71	2.91	0.28	0.644	3.98	− 0.63	2.66	0.27	0.681
6 h	4.30	− 0.72	2.93	0.27	0.664	3.99	− 0.66	2.79	0.27	0.694
12 h	4.34	− 0.66	3.07	0.25	0.647	4.12	− 0.62	2.98	0.25	0.698
24 h	4.75	− 0.65	3.53	0.21	0.666	4.68	− 0.63	3.50	0.21	0.695
48 h	4.56	− 0.59	3.47	0.19	0.712	4.54	− 0.58	3.47	0.19	0.729
72 h	4.70	− 0.58	3.70	0.17	0.708	4.72	− 0.57	3.68	0.17	0.725
96 h	4.91	− 0.56	3.86	0.16	0.699	4.90	− 0.55	3.80	0.16	0.699
Exp	4.98	− 0.69	3.57	0.23	0.664	4.79	− 0.66	3.53	0.23	0.704
(b) *NLS*										
4 h	0.72	0.03	2.68	0.47	0.650	0.16	0.10	2.46	0.63	0.506
6 h	0.35	0.04	4.88	0.71	0.579	− 0.49	0.25	1.08	0.41	0.500
12 h	0.04	− 0.01	2.02	0.27	0.500	− 0.50	0.12	1.51	0.33	0.500
24 h	− 0.09	− 0.09	2.00	0.17	0.500	0.06	− 0.08	2.12	0.16	0.501
48 h	0.24	− 0.15	2.91	0.24	0.564	0.65	− 0.17	3.40	0.23	0.726
72 h	− 0.06	− 0.11	2.35	0.29	0.500	0.69	− 0.21	3.96	0.38	0.686
96 h	− 0.11	− 0.09	1.93	0.25	0.500	0.32	− 0.17	2.91	0.35	0.592
Exp	− 0.01	− 0.04	2.03	0.22	0.500	− 0.38	0.02	1.88	0.25	0.500

Except at MIT = 4 h, *BI*-derived $\alpha$ ($E-D$ thresholds) increase with MIT (Table 5)—with roughly an 18% difference from minimum to maximum under both triggering event definitions. In contrast, *NLS* derived $\alpha$ ($E-D$ thresholds) show no clear trend with MIT, with 77% and 90% variation (excluding MIT = 72 h and 96 h) from minimum to maximum under Peak and End methods, respectively. Moreover, for a given MIT (in $E-D$ space), the *BI*-derived $\alpha$ values vary only slightly between Peak and End methods, whereas *NLS*-derived $\alpha$ values change markedly. This relatively small variation in *BI*-derived $\alpha$ values indicates that *BI* provides more robust and consistent threshold estimates than *NLS*. Furthermore, the systematic trend in *BI*-based $\alpha$ across MIT and its relative stability between triggering event definition suggest that $E-D$ thresholds derived by *BI* depend primarily on sample size rather than variations in triggering event data. In contrast, the large fluctuations in *NLS*-derived $\alpha$ ($E-D$ thresholds) across MIT, despite a constant sample size, imply that *NLS*-derived $E-D$ thresholds are heavily influenced by the specifics of the triggering events themselves.

Unlike the *BI* approach, *NLS* failed to produce reliable $I-D$ thresholds (Table 6). In this study, the *NLS* method fits a power law function to triggering rainfall conditions and establishes a threshold using a 5% non-exceedance criterion. To accommodate the variation in triggering-event intensities, the 5% non-exceedance curve in $I-D$ space has to translate downward, resulting in unrealistically low thresholds that classify nearly all observed rainfall conditions as exceeding the threshold (e.g. MIT = 12 h or MIT = 24 h under the Peak method in Fig. 11). Furthermore, $\beta$ estimates are often extremely small or even positive (Table 6), producing almost flat thresholds (Fig. 11). This behaviour is unrealistic given that intensity should decrease with increasing duration. Since *NLS* provides a single deterministic threshold curve, when the residuals are highly scattered, it struggles to capture the underlying relationship, i ndicating that a simple power-law function may be unsuitable for the $I-D$ under these conditions.

The confidence intervals (CIs) of $\alpha$ in *BI*-derived $E-D$ thresholds (Table 5), marginally increase with MIT under both Peak and End methods, which is consistent with Iadanza et al. (2016) who noted that parameter uncertainties grow as sample size decreases. In contrast, *BI* CIs for $\beta$ narrow with increasing MIT in $E-D$ space for both Peak and End methods. Due to the smaller sample size (n = 52), *NLS* estimated parameters exhibit notably wider confidence intervals, which also vary considerably across MIT and between Peak and End methods, implying that errors in triggering event data may contribute to these uncertainties.

At each MIT, the CIs of both methods are consistently wider for $I-D$ thresholds (Table 6) compared to $E-D$ thresholds, despite similar sample sizes. This discrepancy likely stems from differences in how the rainfall events are distributed in the two parameter spaces. In $E-D$ space, events cluster more predictably along a 1:1 relationship, while in the $I-D$ space, rainfall events appear more dispersed with no well-defined trend. Nevertheless, both threshold spaces exhibit similar trends in CI variation across MIT, reinforcing the influence of the underlying data structure on threshold model uncertainty. These findings highlight the challenges of using $I-D$ thresholds for defining landslide-triggering conditions and suggest a potential advantage of $E-D$ thresholds in providing more stable parameter estimates.

#### Evaluation of threshold model performance

All evaluation metrics vary with MIT, indicating that model performance depends on the chosen MIT. In the $E-D$ space, the *BI* method demonstrates clearer increasing (TPR and FPR) or decreasing (FNR) patterns from MIT = 4 h to 48 h. Rates of change in these metrics from MIT = 48 h to 96 h are insignificant (Fig. 12a, b). At a given MIT, these measures (of *BI*-derived $E-D$ thresholds) remain consistent or show only slight variations between the Peak and End methods. In contrast, *NLS* exhibits greater fluctuations in evaluation metrics both across MITs and between Peak and End methods, underlining the robustness of the *BI* approach and implying that *NLS* is less suitable for analysing threshold model variability across MIT criteria. Similar to $E-D$ space, metrics for $I-D$ thresholds (Fig. 12c, d) display clear trends across MIT with slight differences between Peak and End methods. When using the exponential method and *BI* both $E-D$ and $I-D$ spaces demonstrated performances that constantly fell between those derived using MITs of 24 h and 48 h.

In both $E-D$ and $I-D$ spaces (Fig. 12), *BI*-derived thresholds display a decreasing FNR and an increasing TPR as MIT increases—except at MIT = 6 h under the Peak method in $I-D$ space—indicating a tendency to capture more triggering events as MIT increases, reducing false negatives at higher MITs. However, for *BI*-derived $E-D$ thresholds, FPR increases with MIT (Fig. 12a, b). This implies that while only fewer triggering events are missed by *BI*-derived $E-D$ thresholds, more non-triggering events are misclassified as triggering events at higher MITs. Although TPRs are slightly higher in $I-D$ thresholds (Fig. 12c, d), FPRs are also elevated compared to *BI-*derived $E-D$ thresholds, indicating that $I-D$ thresholds are more cautionary as they have less missed landslides (FN), at the expense of more false alarms (FP). Consequently, the $E-D$ thresholds were more effective than $I-D$ thresholds in reducing false alarms. This behaviour is broadly consistent with the findings of Abraham et al. (2021), who also found that $I-D$ thresholds generated more FP than $E-D$ thresholds. The upward FPR trend in $E-D$ space appears driven by the clustering of larger $E,D$ combinations at greater MIT (Fig. 10). By contrast, no equivalent clustering is evident in $I-D$ space (Fig. 11), resulting in more stable FPR. Overall, the variations in evaluation metrics are more pronounced in $E-D$ space, suggesting that $E-D$ thresholds are more sensitive to changes in MIT than $I-D$ thresholds.

FNR and TPR results show that both *NLS*-derived (except for Exp) and *BI*-derived $E-D$ thresholds detect triggering events more effectively than Melillo ’s (2018) $E-D$ threshold. Similarly, the *BI* updated $I-D$ thresholds also outperform Guzzetti’s (2007) $I-D$ threshold. These comparisons demonstrate the benefit of local recalibration of global/regional models. Furthermore, the overall performance (measured in terms of TSS and ROC-AUC) of the initial $I-D$ (Guzzetti et al. 2007) and $E-D$ (Melillo et al. 2018) thresholds also show an upward trend, though TSS values remain below 0.5 in all cases (initial and updated). This indicates limited discriminatory power and highlights the potential impact of dataset imbalance on ROC-AUC i.e., it can be influenced by the dominant class (non-landslides).

Despite these limitations, the primary goal is to determine the MIT that best represents the rainfall conditions leading to landslide initiation. In both $E-D$ and $I-D$ spaces, the FNR, TPR, and FPR of *BI*-derived thresholds show distinct trends (increasing or decreasing) up to MIT = 48 h, and stabilise between 48 and 96 h, indicating a performance plateau. This suggests that the threshold model performance does not significantly improve beyond MIT = 48 h. Furthermore, across both $E-D$ and $I-D$ spaces, the overall predictive measures—TSS and ROC-AUC—uniformly exhibit a peak at MIT = 48 h, reflecting the best overall model performance. Likewise, *NLS*-derived thresholds, despite some fluctuations, perform best at MIT = 48 h. Additionally, at MIT values of 72 h and 96 h, the evaluation metrics for the NLS-derived thresholds reach extreme values (0 and 1) due to negative $\alpha$ coefficients, which cause all events to lie above the threshold line. To conclude, MIT = 48 h can be considered the most representative duration for separating rainfall events in the study area.

Across both parameter spaces, updated and initial thresholds produce slightly better FPR, TPR and TSS under the End method compared to the Peak method (Fig. 12), whereas FNR is marginally lower. However, the differences observed in evaluation metrics between the Peak and End methods are marginal and statistically insignificant. These results contract slightly with Iadanza et al. (2016), who claimed that the End method tended to underestimate $I-D$ thresholds relative to the Peak method. Although the End method consistently yields marginally better predictive performance in this study, the limited number of landslide events means that a larger dataset or a physically-based approach is needed to definitively determine how best to define the end of a triggering event.

## Conclusion

This study assessed the influence of MIT criteria and triggering event definitions on rainfall threshold estimation for landslide prediction considering both $E-D$ and $I-D$ spaces. To establish thresholds, Bayesian inference (a probabilistic updating procedure) and *NLS* (a nonlinear regression approach) were employed. The performance of derived thresholds against global models from the literature was evaluated using confusion matrix-based measures: FNR, TPR, FPR, TSS and ROC-AUC.

To summarise the key findings of this study, the following conclusions can be drawn:

- The choice of MIT strongly influences rainfall event characteristics and landslide threshold performance. Longer MITs (e.g., 24 h and 48 h) were able to better capture prolonged moisture accumulation (antecedent conditions), providing a more comprehensive representation of pre-failure conditions.
- The exponential method emerged as a robust alternative to manually defined MIT criteria and displayed the second-best performance in terms of all evaluation metrics across the board. Importantly, as a data-driven method, it does not require expert knowledge of rainfall patterns or hydrology of the study area for rainfall event separation.
- Defining triggering events in terms of the End method exhibited slightly better predictive performance overall when compared with the Peak method, however the difference between End and Peak methods was minor.
- BI-derived thresholds were less sensitive to data scatter and more consistent across all MIT criteria and between triggering event definitions (Peak and End) compared to the NLS-derived thresholds, which produced near-flat, unrealistically low threshold curves in $I-D$ space. The robustness of BI makes it better suited for deriving thresholds under varying conditions, including when using the exponential MIT approach.
- The predictive power of *BI*-derived thresholds improved consistently with increasing MIT up to 48 h, beyond which further gains were negligible. It should be highlighted that even these noticeable improvements were achieved at the cost of a higher number of false alarms. Notwithstanding the variations across MITs under *NLS* method, both *BI* and *NLS* consistently demonstrated their best overall predictive performance at MIT = 48 h, confirming it as the most representative criterion for antecedent rainfall conditions in the study area.
- $E-D$ thresholds demonstrated notable advantages over the $I-D$ thresholds, as illustrated by significantly lower false alarms, narrower confidence intervals for model parameters, and clearer trends with increasing MIT.

Overall, this study underscores the importance of methodological choices in landslide threshold modelling. Notably, given the highly imbalanced dataset (far more rainfall events than triggering events), empirical threshold models exhibited poor TSS values, suggesting limitations in their predictive capability in regions with scarce landslide data. This can be overcome by using larger datasets with the *BI* approach or by integrating physically based approaches with statistical methods to achieve improved and reliable landslide predictions.

## Data Availability

The authors are willing to make the code and data used in the project available under reasonable request.
